# Wearable Health Technology to Quantify the Functional Impact of Peripheral Neuropathy on Mobility in Parkinson’s Disease: A Systematic Review

**DOI:** 10.3390/s20226627

**Published:** 2020-11-19

**Authors:** Marta Francisca Corrà, Elke Warmerdam, Nuno Vila-Chã, Walter Maetzler, Luís Maia

**Affiliations:** 1 Instituto de Ciências Biomédicas Abel Salazar (ICBAS), R. Jorge de Viterbo Ferreira 228, 4050-313 Porto, Portugal; luis.filipe.maia@chporto.min-saude.pt; 2 Centro Hospitalar e Universitário do Porto (CHP), Largo do Prof. Abel Salazar, 4099-001 Porto, Portugal; nunovilacha@hotmail.com; 3 Department of Neurology, Christian-Albrechts-University, Christian Albrechts-Platz 4, 24118 Kiel, Germany; e.warmerdam@neurologie.uni-kiel.de (E.W.); w.maetzler@neurologie.uni-kiel.de (W.M.); 4 Instituto de Investigação e Inovação em Saúde da Universidade do Porto i3S, R. Alfredo Allen 208, 4200-135 Porto, Portugal

**Keywords:** peripheral neuropathy, Parkinson’s disease, wearable health technology, functional assessment

## Abstract

The occurrence of peripheral neuropathy (PNP) is often observed in Parkinson’s disease (PD) patients with a prevalence up to 55%, leading to more prominent functional deficits. Motor assessment with mobile health technologies allows high sensitivity and accuracy and is widely adopted in PD, but scarcely used for PNP assessments. This review provides a comprehensive overview of the methodologies and the most relevant features to investigate PNP and PD motor deficits with wearables. Because of the lack of studies investigating motor impairments in this specific subset of PNP-PD patients, Pubmed, Scopus, and Web of Science electronic databases were used to summarize the state of the art on PNP motor assessment with wearable technology and compare it with the existing evidence on PD. A total of 24 papers on PNP and 13 on PD were selected for data extraction: The main characteristics were described, highlighting major findings, clinical applications, and the most relevant features. The information from both groups (PNP and PD) was merged for defining future directions for the assessment of PNP-PD patients with wearable technology. We established suggestions on the assessment protocol aiming at accurate patient monitoring, targeting personalized treatments and strategies to prevent falls and to investigate PD and PNP motor characteristics.

## 1. Introduction

Parkinson’s disease (PD) is a chronic and progressive neurodegenerative disorder, clinically defined by the presence of resting tremor, rigidity, and bradykinesia [1]. These features are collectively referred to as motor symptoms and mostly related to loss of dopaminergic neurons in the pars compacta of midbrain substantia nigra. Alpha-synuclein-positive intracytoplasmatic inclusions, known as Lewy bodies, are the pathological hallmark of the disease [2]. As the disease progresses, motor disturbances represent considerable illness burdens. Deficits in balance and gait are common and disabling features that significantly increase the patient’s risk of falling [3] and the managing of daily living activities [4]. 

PD is also characterized by strong clinical and neuropathological evidence of systemic involvement. The presence of Lewy bodies in several other nervous structures, such as the nervous fibers in the skin, indicate that peripheral nervous system (PNS) involvement may be an intrinsic part in the PD pathological process [5,6]. Since the PNS is a target of alpha-synuclein deposition, it is plausible that intrinsic pathogenic features of PD may predispose to peripheral neuropathy (PNP). 

PNP refers to any disorder of the PNS including single and multiple mononeuropathies, symmetrical involvement of nerves (polyneuropathies), or isolated involvement of sensory ganglia (ganglionopathies) [7]. It usually starts gradually and presents in the most common types a distal-proximal gradient, affecting first the feet and later the hands [8].

The occurrence of PNP in PD (PNP-PD) has been shown to be present in up to 55%, compared to 8% in the general population with comparable age [9,10,11]. Typical features of PNP include postural instability, muscle cramps, and numbness, of which the latter two are more prominent at distal part of the legs. As both PD and PNP pathologies are associated with these symptoms, the concurrence of peripheral involvement could be considered as an additional cause of motor deficits and general worsening in PD [12]. 

PNP can worsen the global functional mobility of patients, since neuromuscular factors (hip strength, ankle proprioception, and decreased peripheral sensation) have been linked to gait and balance difficulties [13]. It is, therefore, plausible to hypothesize that PD patients with PNP (PNP-PD) may develop more prominent gait and balance deficits and, consequently, be at risk of falling, injuries, and reduced quality of life [14]. 

Wearables are constituted of all mobile devices worn on the body (also called on-body sensors), such as inertial measurement units (IMUs), smartwatches, or Holter electrocardiogram monitors [15]. They provide objective and quantitative measures from controlled and unsupervised environments, allowing the development of accurate treatment plans and disease monitoring. In particular, data obtained from IMUs can successfully estimate spatial-temporal parameters and provide sensitive and objective information about motor deficits of various neurological pathologies, which nontechnological motor assessments often cannot identify. Mobility assessment with wearable health technologies are widely investigated in a variety of illnesses, particularly in PD, and allows high sensitivity, accuracy, and reproducibility [16]. However, these methodologies are scarcely studied and have yet to be explored in PNP [17], although a small number of previous works using wearable sensors have successfully demonstrated motor and physical activity characteristics in PNP compared to controls [18,19]. Since the presence of PNP has only recently been considered related to PD, we were interested in understanding whether PNP-PD patients showed specific motor deficits, which can be measured with the use of wearable health technology. For such purpose, a preliminary review of literature performed by the authors showed no studies evaluating the functional impact of PNP in PD on mobility using wearables. Identifying specific gait and balance patterns in this specific subset of PNP-PD patients could provide additional information about gait and balance problems, which can be used to monitor and stratify patients, optimize treatment, prevent falls, and increase quality of life.

For this purpose, in this systematic review we investigated the methodologies (type, number, and location of wearables) mostly used and which parameters (or change of parameter) are the most relevant and clinically useful to characterize PD- and PNP-associated gait and balance deficits. Because of the lack of studies investigating gait and balance impairments in PNP-PD patients with wearables, we divided the search into two parts: We performed a systematic review on the assessment of PNP with wearable health technology and, separately, we reviewed the literature to characterize the use of wearables for PD. The authors defined the major results and conclusions from both searches (PNP and PD) based on the occurrence, significance, and clinical relevance in the included studies. Future directions for the assessment of PD patients with and without PNP phenotype with wearable health technology were then proposed. This study will help to accurately stratify and monitor PD- and PNP-associated functional deficits of gait and balance and target strategies to prevent falls. This could have an impact on the diagnosis and on the clinical approach of PD patients.

## 2. Materials and Methods

### 2.1. Search Strategy

In this systematic review we adopted PRISMA (Preferred Reporting Items for Systematic Reviews and Meta-Analyses) statement methodology [20].

Pubmed, Scopus, and Web of Science electronic databases were searched in April 2020 to identify relevant papers based on their title and abstract. A combination of MeSH (Medical Subject Headings) terms and keywords were used in the search. Since the presence of PNP has only recently been considered related to PD, we were interested in understanding whether PNP-PD patients showed specific motor deficits, which can be measured with the use of wearable health technology. However, a preliminary review of literature performed by the authors showed no studies evaluating the functional impact of PNP in PD on mobility.

Therefore, because of the lack of papers on PNP-PD with wearables, two separate search strategies were used to find relevant papers:

(1) To investigate the main characteristics and the most relevant gait and balance features for studying PNP with wearable technology, the following keywords were used: “peripheral neuropathy” OR “polyneuropathy” OR “small fiber neuropathy” AND “wearable sensor” OR “wearable” OR “mobile health technology” OR “technology assessment” OR “body-worn sensors” OR “inertial sensor” OR “inertial measurement unit” OR “acceleromet*” OR “gyroscope” AND “mobility” OR “gait” OR “balance” OR “postural balance” OR “postural stability” OR “postural strategies”.

In addition, due to the lack of data on PNP with wearables, we performed a literature research to report, narratively and not systematically, other systems, tools, and relevant features coming from other movement analysis methods, used for the assessment of gait and balance in PNP.

(2) To investigate the main characteristics of wearable sensor assessments, and the most relevant gait and balance features in PD, the following keywords were used: “Parkinson” AND “wearable sensor” OR “wearable” OR “mobile health technology” OR “technology assessment” OR “inertial sensor” OR “inertial measurement unit” OR “acceleromet*” OR “gyroscope” AND “mobility” OR “gait” OR “balance” OR “postural balance”.

Unlike in PNP, wearable technology in PD is highly investigated. For this reason, we decided to select the already existing reviews from this search, to provide an overview of PD assessments with wearable technology. The completed search queries are provided in the Appendix A. 

### 2.2. Selection Criteria 

**Figure 1:**
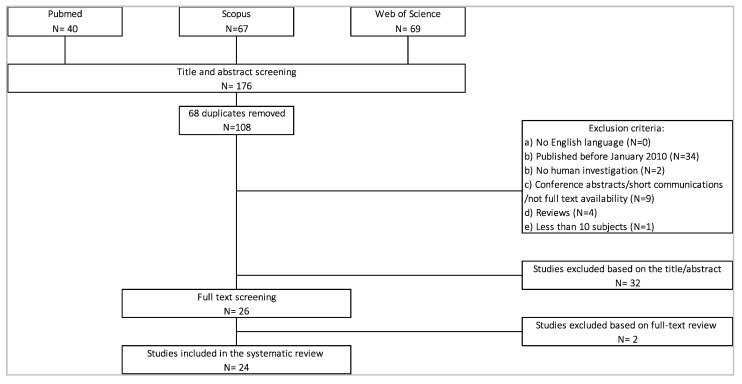
PRISMA (Preferred Reporting Items for Systematic Reviews and Meta-Analyses) flowchart for peripheral neuropathy (PNP) and wearable technology assessment.

**Figure 2:**
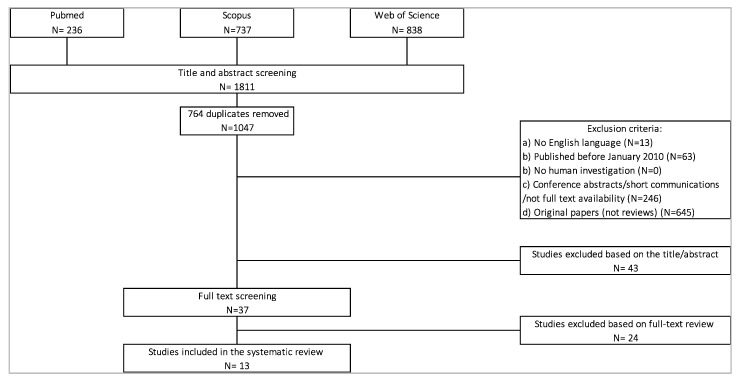
PRISMA (Preferred Reporting Items for Systematic Reviews and Meta-Analyses) flowchart for Parkinson’s disease (PD) reviews.

Research methodology for study selection, according to the PRISMA statement, are shown in Figure 1 and Figure 2. Studies were excluded if they were: (1) not published in English, (2) published before January 2010, (3) not done in humans, (4) nonoriginal full-text manuscripts, (5) a case study or did not enroll >10 subjects, and (6) were out of topic with respect to the aims of the present study (i.e., not regarding PNP, focusing on the validation of algorithms or on machine learning classification, not investigating gait and balance characteristics and parameters, or studying other types of wearables).

Only original papers were considered for the first literature search. For the second part about PD, reviews that were found with the above search criteria were screened. 

After the definition of the selection criteria by all the authors, the selection process was performed by one author. Doubts were decided consensually by three authors.

Works prior to 2010 were not included because wearables were scarcely used for assessing PNP mobility before this date and, secondly, we aimed to focus on the most accurate technology and software, which was mostly developed in this last decade. 

In this work, wearables include all the on-body fixed sensors (tightly fixed to the body with straps, Velcro, or tape) that incorporate at least an accelerometer, gyroscope, or magnetometer or a combination of those and that can extract mobility-related parameters that have been mostly used in research and clinical trials. Ambient sensors were not included in the search because they are not yet commonly used to measure mobility. Therefore, there was not enough literature available to provide any well-founded conclusion about the use of these sensors.

### 2.3. Data Extraction

Upon manuscript selection, the following information was extracted and collected: the type and number of participants and socio-demographic characteristics, the type and location of the wearable sensor(s) used, the main extracted features and the major findings of the study. 

## 3. Results

For the PNP search part, an initial database search identified 176 studies that were potentially eligible for inclusion in this review. After duplicates were removed, 108 abstracts were screened. From these, 26 full texts were selected, of which 24 studies were included in this review (Figure 1). 

For the PD search part, a total of 1811 studies were extracted by the search detailed above. The screening of titles and abstracts removed 1774 studies due to previously stated exclusion criteria. The remaining 37 selected reviews were screened in their full-text versions to assess their inclusion in the review. Finally, 13 reviews were included in this study (Figure 2). 

A summary of the main characteristics of the included PNP papers and PD reviews are reported in Table 1 and Table 2. 

### 3.1. Sample Population Characteristics

Sample population characteristics and sizes varied across the included studies on PNP. The subjects enrolled in these studies consisted of healthy adults (with mean age between 24 and 78 years) and PNP patients with the following etiology: diabetic peripheral neuropathy (DPN) (70.8%), chemotherapy-induced peripheral neuropathy (CIPN) (12.5%), combined DPN and CIPN (8.3%), chronic inflammatory demyelinating polyneuropathy (CIDP) (4.2%), and lower-limb PNP without specific etiology (PNP-LL) (4.2%). Sample sizes ranged from 19 to 434 subjects. 

With regard to PD sample characteristics, the selected reviews described a wide range of participants: for free-living recording at home or home-like environment, sample size ranged between 1 to 467 participant(s) and the majority (49%) of studies were between 10 and 49 participants [21,22,23]. For the lab assessments, the majority of the studies ranged from 5 to 67 participants and four reviews reported studies over 100 study participants.

### 3.2. Sensor Type and Placement

#### 3.2.1. PNP

Multiple wearable sensor types were used within the included articles to assess measures of gait and postural stability in PNP patients. Among the 24 included articles, the most commonly used inertial sensors included a tri-axial accelerometer and a tri-axial gyroscope (83.3% of the studies): LegSys™ and BalanSens™ (BioSensics), used, respectively, for gait and balance assessment; the Opal v1 (APDM) and the Physilog^®^ (BioAGM) for balance assessment; the GaitMeter™ for gait assessment; and the mHT (mHealth Tecnologies) for both gait and balance assessment. Accelerometers only were used in two studies: PAMSys™ (BioSensics) and DynaPort Mini-Mod (McRoberts BV). One study used a gyroscope-based sensor (SwayStar device, Balance International Innovations GmbH) for balance assessment [24]. Sampling frequencies between 50 and 200 Hz were used to acquire the signals. The most commonly used sampling frequency was 100 Hz. 

Several sensor placements and numbers of wearable sensors were used, depending on the task and on the type of assessment. Among the 16 included studies analyzing gait in PNP, four papers (25%) used one sensor, four studies (25%) analyzed gait with sensors on both shanks (two sensors), one paper (6.25%) used four sensors, and six studies (37.5%) assessed gait with five wearable sensors placed on thighs, shanks, and lower back. One study did not report sensor placement (6.25%). 

**Figure 3:**
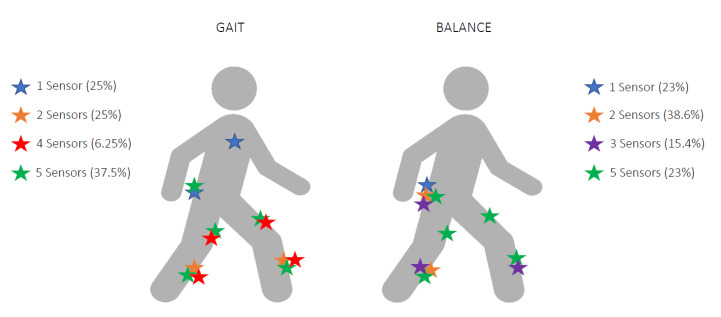
Anatomical representation of sensor placement for gait and balance assessment in patients with polyneuropathy (PNP).

Postural stability was assessed in 13 studies: Three studies (23%) used one sensor on the lower back, five studies (38.6%) used two sensors, and two studies (15.4%) used three sensors on both shanks and lower back. The remaining three studies (23%) utilized five sensors (Figure 3, Table 1).

#### 3.2.2. PD

There is currently no consensus available on the optimum number and placement of sensors to measure PD symptoms. All reviews included that evaluated sensor number and placement showed that the majority of the studies used one sensor placed on the lower back (at lumbar vertebrae level L3, L4–L5, sacrum, or waist) or on the dominant lower limb (thigh, shank, ankle, or foot). Single sensors seemed sufficiently robust for all applications: For gait assessment at home, one sensor was used in 28% to 47% of the studies [21,22,23], while for gait evaluation in the laboratory it ranged from 44% to 69% [25,26]. Not surprisingly, for balance assessment the use of one sensor, and specifically on the lower back, was preferred in 77% to 100% of the studies included in the reviews [26,27,28]. Other most commonly used sensor placements for PD were on both wrists or lower limbs (in 30% of studies) or on lower back and both lower limbs (in 14% of studies) for the home assessment and at both lower limbs (8% of the studies) for laboratory assessment (Table 2). 

### 3.3. Parameters and Main Outcomes

#### 3.3.1. PNP

We included 24 original full-text manuscripts: Eleven studies (45.8%) investigated gait, eight (33.4%) analyzed balance, and five (20.8%) evaluated both gait and balance in PNP patients. 

Gait was assessed mainly during a straight walking task at preferred gait speed, with a distance varying from 7 to 50 m. In two studies patients were asked to perform a 90° turn during walking [29,30]. Several parameters were calculated from the signals acquired through the wearable sensors. The most commonly reported parameters computed from the filtered signals were spatiotemporal gait parameters: gait speed (m/s), stride and step length (m), stride and step time (sec), number of steps, double limb support time (%), and cadence (steps/min). Coefficient of variation (CV) of gait speed and stride length and time (%) was calculated in eight studies [29,30,31,32,33,34,35,36]. Gait speed initiation, number of steps, and total distance required to reach steady-state walking were studied in four papers [34,35,37,38]. Duration (%) and number of walking bouts were extracted in one study [18]. 

Clinical trials among the included papers did not show any statistically significant changes in the gait parameters when comparing pre- and post-intervention. Najafi [39] analyzed gait differences between intervention and control groups after plantar electrical stimulation in DPN patients and Schwenk et al. [33] evaluated gait after a new interactive training in CIPN subjects. Nevertheless, the effect size of these studies suggested the presence of a moderate to large improvement of cadence and gait speed post-treatment. In contrast, Caronni [40] compared the responsiveness to rehabilitation in a group of PNP patients and found a statistically significant difference in gait speed between groups (*p* = 0.001, Table 1). Spatiotemporal parameters were significantly different between PNP patients and healthy controls only in studies investigating gait under more challenging conditions. Kang et al. [32] described a statistically significant difference between DPN and healthy participants in the coefficient of variation of gait speed and stride length during dual-task gait. De Bruin et al. [41] found significant differences in speed, step length, and cadence when comparing DPN patients during dual-task walking on paved trajectories compared to single-task. Another study by Kang [42] showed improvement in stride velocity, stride length, and double limb support (%) during dual-task and fast walking, compared to single-task, after plantar mechanical stimulation. Differences from controls were found in step time, cadence, and gait speed but not in stride length in a study by Esser et al. [17], and gait speed was also 10% decreased in DPN group compared to controls in a study by Ling et al. [31]. Another important result was pointed out by Najafi et al. [34], who found differences in spatiotemporal parameters only during long distances, especially in gait variability and in double support time, when comparing DPN patients with controls. These differences were more pronounced during barefoot walking. 

Balance and postural stability were investigated through numerous tasks. The most frequently used task in all 13 studies was the double leg stance performed in different conditions:

(1) Position of feet: Standing balance was assessed with feet together in eight (61.5%) studies, feet apart (spaced shoulder width) in two studies (15.3%), and both feet positions in one paper (7.6%), while two papers (15.3%) did not specify the position of the feet. In two studies patients were also asked to perform a semi-tandem position [33,43], while one other study introduced a detailed balance test protocol with single leg stance [24].
(2) Open and closed eyes: Twelve studies (92.3%) analyzed balance with both open and closed eyes, and one study only used eyes-open condition [44].
(3) Foam: Two studies used a foam surface (height 10 cm, density 25 kg/m^3^) to analyze balance [24,43]. The other papers only performed balance tasks on firm surfaces.

Other tools to assess postural stability were clinical tests such as the functional reach test [45]. Functional tests (to investigate functional mobility, addressing both gait and balance characteristics) were performed in three selected studies [40,42,45]. They applied the timed up-and-go (TUG) test. This test was split by Caronni et al. [40] into five subphases, and the duration of each phase was measured, as well as the total TUG test duration. 

The included studies reported multiple outcomes of standing balance and postural stability that were calculated from the signals provided by the wearable sensors (Table 1). Of these outcomes, the most commonly reported measures included center of mass (COM) sway (cm^2^), defined as total sway (in seven studies, 53.8%), and related parameters (anterior-posterior (AP) and medio-lateral (ML) sway (cm)). These parameters were also reported in three studies analyzing gait to investigate balance control during walking and gait initiation [34,35,38]. In addition, ankle sway (deg^2^), hip sway (deg^2^), and COM sway area (m^2^) were calculated in six papers (46.1%). Center of gravity (COG) sway (cm^2^), COG AP, and COG ML (expressed in cm) were calculated in one paper [46]. Other parameters were root mean square (RMS, m/s^2^), trunk acceleration, and trunk jerk (m^2^/s^3^) [40,47]; postural coordination of upper and lower body (defined as the reciprocal coordination between hip and ankle motions) [36]; roll and pitch velocity (deg/sec) and roll and pitch angle (deg) [24]. Further parameters were local (in short time intervals, sec) and central (in long time intervals) control balance strategies [46], and cross-correlation function (CCF) of angular velocity to investigate the coordination of human movements [47]. 

A significant reduction in COM sway area (a parameter of postural sway) was shown by Schwenk et al. [33] and Grewal et al. [48] after an interactive sensor-based balance training and by Yalla et al. [45] after an intervention on postural stability with an ankle foot orthosis. These results were found during balance tasks with open eyes, while, interestingly, no significant reduction was found during closed-eyes condition. In contrast, changes of the parameters COM sway area and ML sway area were significant after a virtual reality intervention with eyes-closed and -open conditions [36]. 

#### 3.3.2. PD

In PD, a multiplicity of parameters derived from inertial sensors could be described. For the purpose of this review, parameters from the upper part of the body (upper limb) were not considered. The included reviews listed a series of most relevant spatiotemporal parameters representative of five domains (pace, variability, rhythm, asymmetry, and postural control), which included stride length, stride velocity, cadence, double support time [49,50], and turning velocity [51] followed by step time variability [26,49] and step height, reaction time, and gait cycle duration [52]. Frequency-based measures were dynamics in trunk movement during gait, turning and smoothness [53], harmonic ratio, amplitude, slope and width of dominant frequency, peak trunk horizontal velocity, and phase coordination index of gait cycle [26]. Number of steps, single versus multiple step response, turning duration, turn-to-sit duration, and sit-to-stand and stand-to-sit time- and amplitude-based measures were reported to be important features to determine gait impairment [52]. In more detail, PD patients have been shown to have slower gait, less foot clearance, smaller step lengths, lower turning velocity, lower cadence, and lower peak trunk rotation compared to controls [49,51]. Turning velocity, cadence, and peak trunk rotation were associated with disease progression [54]. Another important parameter in PD is gait variability, also referred to as unsteadiness and arrhythmicity of stepping [55]. Increased gait variability can be seen throughout the disease, and the magnitude of the variability tends to increase with disease severity [49].

Home assessment may have greater ecological validity and gives a true picture of the burden of disease [15]. Parameters that may be particularly relevant for this assessment type are walking bouts (total number of walking bouts, median number of steps per bout, bout duration), turns per hour during the day, duration of each turn, number of steps per turn, peak and average rotational turning rate, and variability of these measures throughout the day and week [22,23].

Regarding standing balance and postural stability, often used parameters were postural sway velocity, RMS accelerations, and jerk [28]. Parameters that may discriminate most effectively between PD and controls are sway area, sway velocity, jerk index, sway amplitude and range of acceleration signals (time domain), and frequency dispersion and centroidal frequency [27,49] (Table 2). 

All these features are able to differentiate between PD and healthy controls (HC) at early stage [26,49], different PD stages [28], different medication states in advanced PD, and PD progression (in particular sway dispersion and sway velocity) [49]. Postural sway is also a good measure of balance control to be used as a primary outcome for interventions [49]. 

## 4. Discussion

We conducted this systematic review to establish the most appropriate approach targeting the number and placement of wearables and most clinically relevant outcomes to assess PNP-associated gait and balance dysfunction in PD patients. We identified the main findings and highlighted general conclusions and suggestions for further study protocols based on (1) how often the parameter is assessed, or how often the sensor is placed on a specific location, (2) the statistical significance of the parameter in the included studies (compared to a control group), (3) the clinical relevance of the parameter in relation to the main scope of the included studies. To our best knowledge, this is the first review to evaluate the existing evidence on PNP-PD. 

The research on wearable health technology to address PNP characterization is lacking, as demonstrated by the small number of studies found according to the inclusion criteria of this review. Almost all the studies included patients with diabetes mellitus (DM) or patients with cancer undergoing chemotherapy. Both conditions have severe consequences on the peripheral nervous system and affect somatosensory function. In particular, diabetic peripheral neuropathy (DPN) affects up to half of the population with diabetes [32] and chemotherapy-induced PNP (CIPN) afflicts up to 40% of patients suffering from cancer [33]. As PNP is most probably a PD-associated symptom, we investigated the main PNP and PD motor characteristics to guide future studies using wearable technology to consider this phenotype in PD. All studies included in this review aimed to investigate both PNP motor deficits and its contribution to (increased) risk of falling and PNP sensory deficits that lead to inadequate proprioceptive feedback, affecting stability during standing and walking. Therefore, given the impact of sensory nervous system in both gait and balance motor activities, we analyzed both domains, gait and balance. 

### 4.1. Gait and Walking Stability

Numerous abnormalities, including sensory loss (impaired vibration, protective sensation), decreased lower-extremity strength, and alterations in the central nervous system, contribute to impaired gait in PNP [57]. 

Our literature search showed that studies investigated mainly gait aspects in PNP patients: Eleven studies examined gait as major primary outcome, while only five papers assessed balance and postural stability (in addition to gait assessment). An explanation for the preference of gait assessment over balance and posture assessment may be the fact that, especially in DPN, the numbness of the feet is considered a major risk factor for increased deterioration in gait function and walking stability [31]. Moreover, footwear that improves gait has been shown to improve quality of life in PNP patients. 

In terms of sensor placement, the amount and the exact position of sensors should consider expected outcomes, practicality, and ease in reproducing the sensor placement [25]. In the selected studies, we found neither a consensus on the position nor on the number of sensors used to investigate gait: Esser et al. [17] showed that a single sensor has the potential to discriminate DPN patients from controls, but it was generally preferred to place sensors on both lower limbs (on the shanks or thighs or both) together with an extra sensor on the lower back. A setup of more than one sensor was preferred in more than 70% of the selected studies, in contrast to PD setups that prefer a smaller number of sensors, usually involving one sensor on the lower back [58]. Generally, gait assessment in PD is performed with one wearable located as close as possible to the COM (i.e., on the lower back) or on one lower limb. This solution is adopted for two reasons: Firstly, this position can track a large amount of body movements (including gait asymmetry and variability, if the sensor is placed on the lower back) [59] and, secondly, it facilitates and simplifies the use of wearables, reducing the intra- and inter-operator variability. 

We believe that the discrepancy between PNP and PD sensors’ setups could be attributable to the expected outcomes and intrinsic characteristics of both pathologies: In PNP the assessment of gait focuses more on variability, step width, and clearance of the feet and, thus, it makes sense to position sensors on both feet. In contrast, gait evaluation in PD relates more to ”whole body” or axial movements [60]. 

Nowadays, a plethora of physical capability assessments and associated algorithms have been developed for the use of one sensor [59], encouraging the simplification of assessment in PD. Since in specific pathological situations the use of sensors placed on both legs is recommended so that data from both sides can be merged [61] and spatial parameters (such as step length, width, and height) are generally more accurate when calculated with a foot or shank sensors, we support the use of more than one sensor for this specific subset of PNP-PD patients (on the lower back and on the lower limbs) to assess gait. 

Spatiotemporal parameters extracted in the selected manuscripts were not always statistically significant in the analysis of PNP compared to healthy participants’ gait. Overall, these results confirmed that, in PNP, the loss of sensation and the inability of the neuromuscular control system to respond to a challenging environment during walking is stronger when attention is reduced [62]. Gait speed and gait variability [29,30,31,34] demonstrated to have a clear association with falling, resulting in relevant parameters to consider when evaluating PNP gait. This is also corroborated by previous literature showing a significant decrease in quality of spatiotemporal parameters, especially for DPN patients [63]. Lastly, the number of steps and distance to reach steady-state gait in the analysis of gait initiation were found to be an important component to investigate risk of falls in people with PNP [35,37,38]: It has been shown that PNP patients take more and slower steps and a longer distance to reach steady-state gait compared to controls. This is due to a decreased somatosensory function, which directly affects performance in the gait initiation phase, increasing unbalance postural transitions and, consequently, the risk of falls. 

Spatiotemporal and frequency-based measures can discriminate PD patients from controls and may also have some potential as surrogate markers for quality of life and disease severity in PD patients [52]. 

In order to gather all the aspects on gait deficits in PD and to reflect a more true-to-life condition, a large amount of papers on PD motor assessment included functional tests to assess various multifactorial aspects other than gait [53]. An example is the use of the instrumented TUG (iTUG) test, which provides an “overview” of functional mobility by assessing sit-to-stand, straight-walking, turning, and stand-to-sit movements [49]. The use of such tools have been shown to be effective to assess gait in PD [64], while for PNP it was only used in a minority of the papers appraised in this review (N = 3). 

In addition, monitoring patients in a daily-living environment and over continuous time periods can make the assessment feasible and ecological. This approach is widely used in PD [23,65], while for PNP only one of the selected papers used monitoring at home to assess gait performances [18]. 

### 4.2. Balance and Postural Stability

Postural control depends on sensory feedback, which includes visual, vestibular, and somatosensory systems. To maintain balance, the central integration of proprioceptive information from the legs with other sensory information is necessary [57]. Individuals with PNP experience balance impairments during gait and standing position, due to absent sensory responses from the lower limbs. This loss in sensory input generally causes instability in trunk sway in people with PNP, even though balance corrections following perturbations to stance are still initiated [24]. 

Our literature search revealed nine of the included manuscripts investigating static balance and postural stability in PNP and four other studies analyzing both gait and balance abnormalities. 

Static balance tasks comprehended a variety of conditions whose general aim was to detect minimal significant perturbations. The most usual adopted strategy was to reduce the support base, asking the subjects to stand still with feet together (which was the assessment protocol in 70% of the selected papers). This approach was widely used because it is easily understandable, repeatable, and can be simply applied to older patients. Other strategies to challenge balance control, such as tandem or semi-tandem positions or one-legged stance, were rarely used because they are relatively difficult to handle for this type of patient (Table 1). 

Only 15.5% of the studies [24,44] asked participants to keep feet apart (usually shoulder’s width or, more specifically, 10 cm between heels and 15 cm between halluces) during assessment, which is in line with a study by McIlroy and Maki [66], who recommended to avoid ‘unnatural’ or ‘uncomfortable’ foot positions in favor of a preferred foot placement. 

The strategy of open and closed eyes and the use of foams were adopted in order to reduce the remaining contribution of lower leg proprioceptive feedback to balance control and to understand the level of visual cueing in PNP patients. Four studies performed balance tasks barefoot [24,43,46,48], an interesting approach that could be applied to emphasize PNP impairments, even if not always applicable because of neuropathic complications (i.e., diabetic foot ulcerations) [67]. 

In PD, a standard feet position during stance tests is not fully established [27]. When it is preferred to keep the feet apart, because it is a more ecological condition, the performances can be biased by the subjective selection of the base of support. This can lead to contradictory findings due to methodological differences between subjects and studies. To avoid discrepancies, Hubble et al. [28] recommended to stand with eyes open and feet of maximum 10 cm apart during stance tests.

Several ways exist for estimating postural sway. An important rule to consider is to place at least one inertial sensor at the lower back, often the best position to monitor the COM [43], to examine both PNP- and PD-related deficits. A single accelerometer worn on the lower back has been validated to assess balance characteristics [68], but this approach may be not appropriate for assessing postural sway, for example, during large sway fluctuations or reaching task movements [43]. To overcome this defect, using more than one sensor, especially on the lower limbs, is recommended. This is also confirmed by the included studies: Ten of 13 papers used more than only the sensor on the lower back (Table 1). 

Moreover, this is also confirmed in PD assessments: One sensor on the lower back was used to perform posturographic examination, while additional sensors on the lower limbs were preferred to assess (further) postural strategies [27].

Regarding the relevant features for balance and postural stability, interesting conclusions can be made from the included studies of PNP. First of all, compared to healthy controls, COM-AP sway amplitude seems to be associated with the presence of neuropathy symptoms [44,47]. This is in line with evidence from literature: Higher AP sway may be associated with PNP as a result of an increased sway at the hip joint [69]. In fact, healthy individuals rely on the ankle joint to control sway (ankle strategy), while PNP patients predominantly showed a hip strategy, to benefit from more accurate proprioceptive information from receptors at the hips [70].

A second notable result is that COM-ML sway amplitudes are obviously a good predictor of falls. It has been shown that ML sway was associated with falls in PNP patients [42,44]. These data are consistent with other populations, such as elderly [71].

Clinical trials did not find significant differences in postural sway before and after treatment between intervention and control groups. However, the most promising parameter may be ankle sway: This parameter showed the highest effect size (Cohen’s d = 0.76; *p* = 0.001) after plantar electrical stimulation [39]. 

In PD, postural sway in both AP and ML directions was also the most analyzed feature during stance tests [53]. Other relevant parameters of postural stability are jerk index, the range of acceleration signals, frequency dispersion, and centroidal frequency [27]. 

Overall, AP, ML, and total sway frequencies need to be taken into consideration when investigating postural stability in PNP [46] and PD, using both open- and closed-eyes tasks and static and dynamic balance tests [24], in addition to hip and ankle sway (for both hip and ankle strategies). The last was shown to be greater also in CIPN patients during both eyes-open and -closed conditions, suggesting a pronounced visual dependency of PNP for ankle stability [56].

A final consideration to point out is the feasibility of wearables in assessing motor symptoms. Among the included papers on PNP, IMUs’ feasibility and accuracy were investigated by Najafi et al. [43], who compared balance features with center of pressure (COP) measures from a standard pressure platform in a group of healthy subjects and in a group of PNP patients. Results suggested a relatively high correlation (r = 0.92) between the two measurements during all the study conditions, and the same IMUs’ protocol was then used and repeated in other further studies from the same group [18,56]. In addition, the same IMU measures were compared to clinical scores during different conditions (open-eyes and closed-eyes conditions). With regard to PD, IMUs’ accuracy and feasibility were pointed out in the work by Oung et al. [50], who compared this technique with video recording and clinical evaluation (i.e., Unified Parkinson’s Disease Rating Scale - UPDRS scores). Sensitivity and validity of IMUs were also confirmed in the review by Godinho et al. [16]: Reliability was investigated comparing IMUs’ sway with force-plate measures, and test-retest reliability were also confirmed by clinical balance tests. For both pathologies (PNP and PD), we found no information on accuracy and feasibility based on sensor location.

### 4.3. PNP Motor Assessment with Other Tools than Wearables

Clinical scales and complex approaches are noteworthy in the evaluation of PNP functional disabilities, although these tools present disadvantages: They are time-consuming and require specific expertise. In addition, complex tools are reserved only for clinical settings due to their high cost and complexity of technology and can capture only a few steps and often do not represent the full gait complexity. An overview of the main clinical scales and these complex systems is provided in the following paragraphs. 

#### 4.3.1. Gait Assessment

Clinical scales represent reliable and valid measures of disease characterization and monitoring. Worth mentioning in the evaluation of PNP gait disturbances are the functional gait assessment scale, which effectively classifies fall risk and predicts unexplained falls [72], and the Dynamic Gait Index, assessing the ability to adapt gait to complex tasks and walking stability [73]. For their efficacy and sensitivity, these clinical scales are often chosen as primary outcomes in intervention studies. 

More complex equipment was also used to evaluate gait in PNP. The 3D optical motion capture systems measure the position and orientation of corporal segments in space [74] and provide a large amount of gait characteristics that can be investigated. Optical motion capture systems are often combined with force plates: mechanical sensing apparatus designed to measure the ground reaction forces and moments involved in the human movement [75].

The vast majority of studies on gait assessment in individuals suffering from PNP used optical motion capture systems, force plates, or a combination of the two (56.7% of the included papers). Particularly, foot and foot joints were relevant targets in the investigation of DPN. This is due to the fact that PNP is one of the key factors in the pathogenesis of diabetic foot and its chronic complications [76].

Hip abductors’ range of motion or hip angles, knee flexion, ankle joint dorsiflexion, and metatarso-phalangeal flexion-extension were the focus of investigation of gait patterns in PNP with motion capture analysis [76,77,78,79,80]. Differences were found in spatiotemporal parameters during walking on smooth and uneven surfaces in DPN [81], while a significant increase was found in toe clearance [78,82] and step width [76,83] of PNP patients compared to controls. Other relevant features analyzed were foot rotation on the sagittal plane, knee and ankle strength [84], dorsal and plantar flexors strength [85], dynamic plantar pressure at the forefoot [86], and peak forces of ankle (flexors, extensors, and evertors) [77]. 

Another frequent tool (in 19.4% of the included papers) in the examination of PNP gait was the use of electronic walkways. These electronic walkways are pressure-sensitive carpets (the most used was the GAITRite^®^ system), a computerized walkway system for the quantification of spatiotemporal gait parameters. They are portable and embedded with pressure sensors that detect a series of footfalls [87]. Electronic walkways were used for the analysis of gait in PNP subjects to study treatment effects [88], to characterize PNP global gait [89], to investigate the functional impairment in daily activities [90], to study cognitive deterioration during dual-task condition [91] and to analyze gait patterns at different locomotion speeds [92]. 

#### 4.3.2. Balance and Postural Stability

For the examination of balance performances in PNP, the Berg Balance Scale (BBS) was the most used clinical scale [73,93,94,95,96,97,98,99]. BBS is a standard clinical measure to assess static balance impairments and a robust method to study postural control [100]. The Tinetti Balance scale (TBS) is another valid clinical scale to measure balance: Monti Bragadin et al. [99] demonstrated the importance of both TBS and BBS tests in the evaluation of disability in PNP and, in particular, in identifying those patients who present a substantial risk of falling. The Fullerton Advance Balance test (FAB) [101,102,103] is being increasingly utilized because of its capacity to assess postural control among higher functioning independent older adults [104]. Contrary to the BBS, FAB test examines both static and dynamic postural control, sensory reception, and integration and incorporates a secondary task [100]. A few studies utilized the Romberg test to assess postural stability with simple scoring ‘pass or fail’ [19,105]. Participants were classified as having dysfunctional balance if they failed any of the four Romberg test conditions. Although quick and simple, this method cannot define postural stability impairments with accuracy. 

With respect to other approaches, most studies have employed force plates in the evaluation of postural stability (71.4% of the papers included in the narrative search). Force platforms measured the COM projections over the base of support and recorded postural stability in two ways, with static and dynamic posturography. The dynamic approach analyzes postural reactions in response to a translation of the support surface, to the visual surrounding, or both [106].

Static balance assessment was more adopted compared to dynamic posturography (in 64.2% of the included papers) in the investigation of PNP. Static posturography with force plates was used to evaluate the effect of a rocker outsole shoe on postural stability [107] and of a new insole design [108] in individuals with DNP. Manor et al. [109] and Alsubiheen et al. [110] used static balance assessment with force plates to examine the effects of Tai-Chi on standing COP dynamics in adults with PNP, resulting in an increased complexity of standing dynamics and significant improvement after intervention. Force platforms were used to quantify differences in postural stability: to assess the effect of intervention on stability in CIPN survivors [96,111], the impact of a sensorimotor exercise program [103,112], and the influence of a balance and endurance training, which resulted in an improvement in sway path [113].

Changes in body sway were also compared between DNP and Charcot-Marie Tooth subjects, indicating more impaired static control of balance in the DNP group, possibly due to small and large afferent fibers’ involvement [114]. Static balance assessments also allowed evaluating postural control and fall incidence in PNP [115], to assess postural stability in the PNP population on either firm or foam surfaces [116], and to differentiate between PNP and healthy controls [117].

Moreover, static balance was also examined without the use of force plates in five studies (17.8%). McCary et al. [118] used a swaymeter (Neuroscience Research Australia, Sydney) to quantify postural sway pre- and post-rehabilitation in people with CIPN. In another study, sway amplitude and velocity were analyzed through a head and hip electromagnetic tracker [119]. Finally, baropodometric platforms were used in three studies [97,120,121]: These tools use the load and the plantar pressure on the mat to define footprint shape and assess foot deformities and barefoot plantar pressures. 

Dynamic posturography was chosen in the 28.5% of the studies and comprehended the sensory organization test (SOT). During the SOT, subjects are instructed to stand still and maintain balance using the visual, vestibular, and proprioceptive systems. The SOT evaluates patients’ ability to effectively use the three sensory systems to maintain postural stability. In PNP, dynamic balance tests with force platforms were used to evaluate the altered sensory organization during stance [122] and postural sway reactions [123] in CIPN patients. This approach was also chosen to assess standing postural reactions in demyelinating PNP [124] and the effects of PNP in detecting short postural perturbations [125]. A study by Razzak and Hussein [126] highlighted a greater visual dependence in DNP patients faced with postural challenging situations, while Rao and Aruin [127] suggested that auxiliary sensory cues improved automatic postural responses. 

In conclusion, wearable health technology is increasingly becoming an attractive alternative to conventional assessment tools to assess PD, PNP, and PD-PNP patients in clinical routine management and in clinical trials. These novel technologies have greater applicability especially for the assessment of daily life activities and, finally, are cheaper and less complex compared to conventional, lab-based equipment. 

## 5. Conclusions

We consider the use of wearable health technology for the assessment of PNP in PD of great advantage compared to clinical scales and conventional, lab-based assessment tools, as the former allow for more consistent and reliable results.

**Figure 4:**
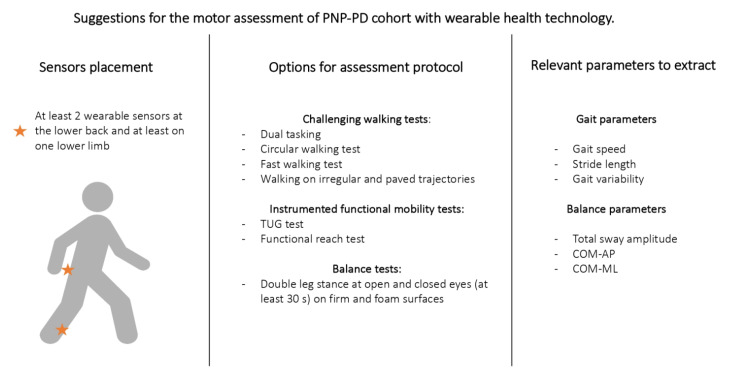
Suggestions for the motor assessment of PNP-PD cohort with wearable health technology.

The following suggestions may help assessing this cohort (Figure 4):

- A combination of at least two sensors (one on lower back and one on at least one lower limb) may help gathering both PNP- and PD-specific features during gait and balance testing.
- Concerning parameters to analyze, particular attention should be given to gait speed, stride length, and gait variability. Gait variability may be particularly relevant for PNP-induced gait changes. Dual tasking assessments and irregular trajectories may unveil PNP-related gait deficits that are not visible during nonchallenging, single tasking walking conditions.
- Functional mobility tests (TUG test, functional reach test) can provide a comprehensive overview of function and mobility in PD patients with and without PNP.
- Balance tasks should include double leg stance with open- and with closed-eyes conditions.
- Total sway amplitude and AP and ML sway directions may be the most promising balance parameters to differentiate between PD and PD-PNP.

Overall, these suggestions may help to accurately stratify and monitor PD- and PNP-associated functional deficits of gait and balance and target personalized treatments and strategies to prevent falls. This could have an impact on the diagnosis and clinical approach of this subset of patients in particular and on the aged population in general. 

## Figures and Tables

**Table 1:** Summary of the major characteristics of the research design, analyses, and outcomes for the studies on PNP that met the inclusion criteria.

REFERENCE	POPULATION (Mean Age ± SD)	SENSORS (Number and Type)	SENSOR PLACEMENT	ASSESSMENT PROTOCOL	PARAMETERS EXTRACTED/INVESTIGATED/OUTCOMES	MAIN FINDINGS
Ling et al., 2020 [31]	12 DPN + DFU (55.6 ± 3) 27 DPN (64.3 ± 1) 47 Healthy controls (62.9 ± 2)	5 Inertial sensors (ACC, GYR and MAG) (LegSys™, BioSensics) Freq: 100 Hz	Thighs Shanks Lower back	Straight walking test at preferred speed for 10 m on a flat floor	Gait speed and gait speed unsteadiness, stride length and stride length unsteadiness, gait cycle time, double support and double support limp, step length limp, gait symmetry	People with DPN and DFUs wearing offloading devices have poorer gait function compared to controls. DFUs and offloading devices further deteriorate gait beyond DPN, specifically for performance in gait speed, stride length and gait cycle time. Compared to controls, DPN showed 10% decreased in gait speed and increased stride length of 48%.
Kang et al., 2020 [37]	38 DPN (72.6 ±5) 33 Healthy controls (77.9 ± 8)	5 Inertial sensors (ACC, GYR and MAG) (LegSys™, BioSensics) Freq: 100 Hz	Thighs Shanks Lower back	Straight walking test at preferred speed for 12 m on a flat floor at two conditions: during single and dual (cognitive) task	Number of steps and distance to reach steady-state gait Gait speed and body sway in the mediolateral direction in the gait initiation phase and steady-state gait speed.	For both single-task and dual-task gait conditions, number of steps, distance, and mediolateral body sway were significantly greater for the DPN group than for the CON group. Gait initiation steps and dynamic balance may be more sensitive than gait speed for detecting gait deterioration due to DPN.
Kang et al., 2020 [32]	44 DPN + CIPN: 25 PNP without cognitive impairment (66.5 ± 9) 19 PNP with cognitive impairment (68.5 ± 9)	2 Inertial sensors (ACC, GYR and MAG) (LegSys™, BioSensics) Freq: 100 Hz	Shanks	Straight walking test at preferred speed for 12 m on a flat floor at two conditions: during single and dual (cognitive) task	Coefficient of variation (CV) of gait speed, stride length and stride time Spatio-temporal gait parameters: gait speed, stride length and stride time	During dual-task walking, between-group differences were significant for gait variability for gait speed and stride length (51.4% and 71.1%, respectively; *p* = 0.014 and 0.011, respectively). The presence of cognitive impairment exacerbates the risk of falls in people with PN.
Kang and Najafi, 2020 [18]	49 PNP (DPN+ CIPN) (68.5 ± 7)	1 accelerometer (ACC) (PAMSys™, BioSensics LLC) Freq: 50 Hz	Chest	48-h period recording	Durations of standing posture Sedentary posture Total number of walking bouts Number of total steps	People with PN and low concern about falling tended to have more activity, but people with PN and high concern about falling tended to have less activity. Furthermore, the duration and amount of being active (i.e., walking bout and total step counts) may predict the level of concern about falling, and thus may be used as eHealth targets and strategies for fall risk assessment among people with PN.
Zahiri et al., 2019 [56]	84 subjects with cancer (CIPN+ and CIPN-) (71.1 ± 9) 57 Healthy controls (69.5 ± 9)	5 Inertial sensors (ACC, GYR and MAG) (LEGSys™and BalanSens™; Biosensics LLC) Freq: not reported	Shanks Thighs Lower back	Gait assessment: single-task (no cognitive distraction) over 15 m at a self-selected speed. Balance: double leg stance 30 s with feet close together during eyes-open and eyes-closed situations.	Gait parameters: stride velocity, stride length, stride time and double support time. Balance parameters: area of ankle sway, area of hip sway, area of center of mass (CoM) sway, and CoM sway in the medial- lateral (ML) direction.	The deterioration in gait parameters was more pronounced in the CIPN+ than the CIPN- subgroup, when compared to the control group. CIPN+ on average had 8% and 18% slower stride velocity compared to the CIPN- and control groups, respectively. Stride velocity was also on average 11% slower in CIPN, when compared to control. Similar trends were observed for other gait parameters of interest. Results also suggest high visual dependency in the CIPN+ subgroup. The negative impact of CIPN on motor-performance is confirmed with the largest effects on ankle stability and stride time. Vibration perception threshold (VPT) is a predictor of motor deterioration and may be used to determine the severity of CIPN symptom
Kang et al., 2019 [42]	30 DPN (68.1 ± 9)	Gait assessment: 5 Inertial sensors (ACC, GYR and MAG) (LEGSys™, Biosensics) Balance assessment: 2 Inertial sensors (ACC and GYR) (BalanSens™, Biosensics) Freq: not reported	Gait assessment: Shanks Thighs Lower back Balance assessment: Dominant leg Lower back	Gait: 10 m walking test at normal and fast pace, and at two conditions: single and dual tasks. Static balance: (i) double leg stance for 30 s with feet together with eyes open and eyes closed (EC). (ii) semi-tandem stance for 30 s Global functional mobility: TUG test	Gait parameters: stride velocity, stride length, stride time and double support time. Balance parameters: area of ankle sway, area of hip sway, area of center of mass (CoM) sway, and CoM sway in the medial- lateral (ML) direction.	Daily use of plantar mechanical stimulation through a micro-mobile foot compression device installed in a shoe insole is effective for improving vibration perception, which likely results in improvements in some balance outcomes and gait parameters. Key findings were improvements in plantar sensation in the foot, CoM sway in the ML direction during quiet standing and stride velocity and other spatiotemporal gait parameters in dual task condition after using the wearable foot compression device for four weeks.
Fino et al., 2019 [44]	216 CIPN+ (63.0 ± 6) 218 CIPN- (62.2 ± 6) 49 Healthy controls (63.3 ± 6)	1 Inertial sensor (ACC and GYR) (Opal v1, APDM) Freq: 128 Hz.	Lower back	Double leg stance test with eyes open for 30 feet apart	AP-sway, ML-sway, or resultant sway	Cancer survivors had worse sway than healthy control subjects in components related to sway magnitude and mediolateral frequency of sway, but no difference in the component related to resultant/AP sway jerk and frequency. Cancer survivors who reported neuropathy were more likely to have higher resultant/AP sway frequencies and jerk than asymptomatic survivors, while survivors who reported a fall were more likely to have lower frequencies of mediolateral sway than non-fallers. Neuropathy influenced the associations between specific characteristics of sway and falls, which may have implications for fall prevention interventions.
Caronni et al., 2019 [40]	25 PNP-LL (76.5 ± 6)	1 Inertial sensor (ACC and GYR) (mHT, mHealth Technologies) Freq: 100 Hz	Lower back	Gait: 10 m walking test and TUG test repeated five times each. Static balance: double leg stance for 30 s with (i) feet apart (FA) and eyes open (EO), (ii) feet apart and eyes closed (EC), (iii) feet together (FT) and eyes open an (iv) feet together and eyes closed.	Gait: 5 subsequent phases of TUG test: sit to stand (STS), walk 1 (W1), turn 1 (T1), walk 2 (W2) and turn and sit (TAS); duration of each phase and total TUG duration (TTD); mean vertical angular velocity during turn 1 and during TAS Root mean square (RMS), trunk acceleration (Trunk acc) and trunk jerk (Trunk jerk).	After rehabilitation, patients with PN-LL consistently improved straight walking, walking along curved trajectories and transfers, with no apparent modification of static balance. Four gait measures (i.e., gait speed, angular velocities during TUG) and the TTD showed a large improvement after rehabilitation. The improvement was medium for the walking phases of the TUG test (i.e., W1, T1 and W2) and TUG transfers (i.e., STS and TAS).
Findling et al., 2018 [24]	11 CIDN (chronic inflammatory demyelinating polyneuropathy) (61.1 ± 11) 10 not inflammatory PNP (68.5 ± 11)	1 gyroscope SwayStar device (GYR) (Balance International Innovations GmbH) Freq: 100 Hz	Lower back	12 stance tasks: 4 double leg tests with the feet spaced shoulder width apart; 4 tasks with eyes open on a normal surface and on a foam surface (height 10 cm, density 25 kg/m3)± and eyes closed. 3 single leg stance tasks with eyes open, 2 on a normal surface (right and left leg) and 1 on the foam surface. 1 task with single leg standing. 5 tasks for dynamic balance: 8 steps tandem gait 3 m walking on heels 3 m walking pitching the head up and down 3 m walking with eyes closed and 8 m walking with eyes open	Global balance control index (BCI); trunk sway and trunk velocity	CIDP patients have reduced ability to decrease trunk sway with lower gait speed. A similar effect was noted for pitch velocity walking eyes closed. This is possibly associated with an increased risk of falls
Esser et al., 2018 [17]	17 DPN (63 ± 9) 42 Healthy controls (61 ± 4)	1 inertial sensor (ACC and GYR). Freq: 100 HZ	Lower back	Gait: 10 m at normal and fast pace	Step time, cadence, stride length, walking speed	A single IMU used in clinical setting has the potential to discriminate patients with DPN compared to healthy controls. Walking speed was the most sensitive parameter, while no significant differences were found in stride length compared to controls.
Najafi et al., 2017 [39]	28 DPN: 17 intervention group (56 ± 5) 11 Healthy controls (64 ± 10)	Gait assessment: 2 Inertial sensors (ACC, GYR and MAG) (LEGSys™, Biosensics) Balance assessment: 2 Inertial sensors (ACC and GYR) (BalanSens™, Biosensics) Freq: not reported	Gait assessment: Shanks Balance assessment: Dominant leg Lower back	Gait: 10 m at normal and fast pace Balance: double stance for 30 s with feet close together (without touching), with eyes open (EO), and eyes closed (EC).	Gait: Stride velocity, stride time, stride length and cadence. Balance: COM anterior-posterior (AP) sway, medial-lateral (ML) sway, and total sway area	No differences were observed between the groups for baseline characteristics or for motor performance including postural sway and spatiotemporal parameters of gait. However, the majorities of measurable metrics were improved post-treatment in the intervention group with no significant changes in the control group. This study suggests that daily home use of plantar electrical-stimulation may be a practical means to enhance motor-performance and plantar-sensation in people with DPN.
Schwenk et al., 2016 [33]	22 CIPN (70.3 ± 8)	Gait assessment: 4 Inertial sensors (ACC, GYR and MAG) (LEGSys™, Biosensics) Balance assessment: 3 Inertial sensors (ACC and GYR) (BalanSens™, Biosensics) Freq: not reported	Gait assessment: Shanks Thighs Balance assessment: Shanks Lower back	Gait: 10 m at normal pace Balance: double stance 30 s with feet close together (without touching), with eyes open (EO), and eyes closed (EC), and semi-tandem position with EO.	Gait: gait speed and variability Balance: COM AP sway and ML sway; hip sway and ankle sway	ML CoM sway, hip sway, and ankle sway were reduced in the intervention group compared to control group during balance assessment with feet close together and EO. Significant reductions in postural sway parameters were also found during the more challenging semi-tandem position, except for ankle sway. Older cancer patients with CIPN can significantly improve their postural balance with specifically tailored, sensor-based exercise training.
Toosizadeh et al., 2015 [46]	18 DPN (65 ± 8) 18 Healthy controls (69 ± 3)	2 Inertial sensors (ACC and GYR) (BalanSens™, Biosensics) Freq: not reported	Ankle Hip	2 Romberg balance trials (with open and closed eyes) for 15 s	Center of gravity (COG) sway (total sway) and COG (AP) sway, COG (ML) sway; local- (in short time-intervals) and central- (in long time intervals) control balance strategies.	The rate of sway within local-control was significantly higher in the DPN group by 49%, which suggests a compromised local-control balance behavior in DPN patients. Unlike local-control, the rate of sway within central-control was 60% smaller in the DPN group, which suggests an adaptation mechanism to reduce the overall body sway in DPN patients. In the lack of sensory feedback cueing, DPN participants were highly unstable compared to controls. However, as soon as they perceived the magnitude of sway using sensory feedback, they chose a high rigid postural control strategy, probably due to high concerns for fall, which may increase the energy cost during extended period of standing.
Grewal et al., 2015 [48]	35 DPN: 19 intervention group (62.5 ± 7) 16 Healthy controls (64.9 ± 8)	5 Inertial sensors (ACC, GYR and MAG) (LEGSys™, Biosensics LLC) Freq: 100 HZ	Shanks Thighs Lower back	Double leg stance for 30 s with open and closed eyes and feet together	COM sway, COM AP, COM ML sway, Hip sway.	On average, the CoM sway area for the intervention group (IG) was reduced significantly by 58.31% compared to a reduction of 7.8% in the control group (CG). The IG showed a significant reduction in the ML CoM sway; similarly, significant reductions were observed for the hip and ankle sway in the IG compared to the CG. During balance assessment with closed eyes, the IG achieved a reduction in CoM sway of 62.68%; however, none of the sway components (AP, ML or CoM area) reached significance. People with DPN can significantly improve their postural balance with diabetes specific, tailored, sensor-based exercise training
Yalla et al., 2014 [45]	30 DPN (73 ± 6)	5 Inertial sensors (ACC and GYR) (BalanSens™, BioSensics LLC) Freq:100 Hz	Shanks Thighs Lower back	6 double stance of 30 s trials (2 for each footwear condition during eyes-open and eyes-closed) with their arms crossed, feet positioned closeto each other without being in contact. Dynamic balance: Functional reach task Global functional mobility: TUG test	Ankle, hip, and COM sway	The orthoses reduced center of mass sway on average by 49.0% and 40.7% during eyes-open balance trials. The reduction was amplified during the eyes-closed trials with average reductions of 65.9% and 47.8%, compared to barefoot and ‘shoes alone’ conditions. Ankle foot orthoses reduced postural sway and improved lower extremity coordination in the elderly participants without limiting their ability to perform a standard activity of daily living.
Karmakar et al., 2014 [29]	19 NeP-DPN (65.7 ± 10)	2 Inertial sensors (ACC and GYR) (GaitMeter™) Freq: not reported	Shanks	Straight walking test at preferred speed for 50 m on a flat floor and a 90° turn without rest time.	Step length, step velocity, gait variability	DPN subjects with neuropathic pain receiving pregabalin treatment had increasing variance for both step length and step velocity. No significant differences in durations of time required to walk, step length and step velocity measures were found between timepoints and interventions. The degree of variability in both step length and step velocity significantly increased for subjects receiving pregabalin for comparison of baseline and final visits. The potential relief of NeP using pharmacotherapy may not improve gait dysfunction.
Najafi et al., 2013 [34]	12 DPN (60 ± 12) 8 Healthy controls (60 ± 6)	5 Inertial sensors (ACC and GYR) (LEGSys™, Biosensics LLC) Freq: not reported	Shanks Thighs Lower back	Straight walking test at preferred speed for 7 m (short distance) and 20 m (long distance) at two conditions: barefoot and with regular shoes.	Gait initiation velocity, stride velocity, gait variability, average range of motion of ML- and AP- CoM during each stride, double support time, stride time, stride length, number of steps.	Most gait parameters showed alterations in patients with DPN during the barefoot and shoe conditions compared with those in the control group. However, the effect size was usually larger in the long walking distance trials, and none of the observed differences were statistically significant in the short walking distance trials. Gait speed during the gait initiation and gait steady state phases was reduced on average by 15%. Variability was 84% higher in the DPN group. Double support time was more than 20% during the barefoot and shod conditions in those with DPN, suggesting a more altered gait while walking barefoot. The benefit of footwear was significant only during the long walking distance trials.
Lalli et al., 2013 [30]	20 DM (60.2 ± 13) 20 DPN (62.6 ± 9) 22 NeP-DPN (63.9 ± 9) 24 Healthy controls (58.8 ± 11)	2 Inertial sensors (ACC and GYR) (GaitMeter™) Freq: not reported	Shanks	Straight walking test at preferred speed for 50 m on a flat floor and a 90° turn without rest time.	Gait variability, cadence, step length, step velocity and total duration of walk	No differences were observed among groups in the total duration of walk, step length and step velocity. The degree of variability in both step length and velocity were both significant in participants with NeP-DPN compared to DPN. Participants with NeP-DPN had greater variance in gait when compared to DPN and controls. Also, patients with DPN or DM only were not significantly different from controls with respect to most gait measures utilized. NeP contributes to gait variability, potentially contributing to the risk of falling in DM patients.
Kelly et al., 2013 [35]	16 DPN (73 ± 8) 18 DM (62 ± 8)	5 Inertial sensors (ACC, GYR and MAG) (LEGSys™, Biosensics LLC) Freq: not reported	Shanks Thighs Lower back	Straight walking test at preferred speed for 20 m on a flat floor	Gait: stride velocity, stride length, stride time, double support time, gait speed variability, steps required to reach steady-state walking, AP and ML COM sway during walking	Gait performance was relatively worse in participants with DPN compared with DM individuals. However, only steps taken during gait initiation and double-support percentage achieved statistical significance. The DPN and non-DPN groups had almost the same level of concern about falling, suggesting a prevalence in older adults with DM but not a relation with DPN.
Grewal et al., 2013 [36]	29 DPN (57 ± 10)	2 Inertial sensors (ACC and GYR) (BalanSens™, BioSensics LLC) Freq: 100 Hz	1 Shank Lower back	Double stance position for 30 s at open and closed eyes (width not specified)	COM sway (AP and ML) and sway area. Postural coordination between the upper and lower body (in the mediolateral and anteroposterior directions)	Significant reduction in center of mass sway after training. A higher postural stability deficit (high body sway) at baseline was associated with higher training gains in postural balance (reduction in center of mass sway). In addition, significant improvement was observed in postural coordination between the ankle and hip joints.
Grewal et al., 2013 [38]	16 DPN + DFU (58.3 ± 4) 15 DPN (54.2 ± 11) 8 Healthy controls (59.6 ± 6)	A set of Inertial sensors (LEGSys™, Biosensics LLC) (ACC and GYR) Freq: not reported	Not reported	Not reported	Stride velocity, stride length, gait cycle time, double support time, AP- and ML- COM sway area, knee range of motion, gait variability, number of steps and total distance required to achieve gait steady state	During gait initiation, number of steps, knee range of motion and CV stride velocity revealed significant differences among groups. The presence of PNP increases the number of steps required to reach steady state gait by nealry 90% compared to healthy individuals. During steady state gait, double support, COM sway area anf CV stride velocity were significantly different between groups. The reuslts demonstrates that neuropathy deteriorates gait, but the presence of foot ulcers does not alter gait parameters further than neuropathy. In addition, patients with foot ulcers demonstrated a better gait compared with DPN patients without ulcers.
Turcot et al., 2012 [47]	25 DPN (63.5 ± 7)	3 Inertial sensors (ACC and GYR) (Physilog^®^, BioAGM). Freq: 200 Hz	Shanks Lower back	Double leg stance for 30 s with open and closed eyes (width not specified)	Angular velocity at trunk and ankle levels in two terms: RMS and with cross-correlation function (CCF), to investigate the coordination of human movements in motor control. CFF was calculated between trunk and right ankle, trunk and left ankle, right and left ankle.	The analyses of anterior-posterior angular velocities between the trunk and both ankles showed positive CCFs in the eyes open condition in 23/25 patients and in all patients in the eyes closed condition. It has been demonstrated that the level of PNP was linked to postural strategies and instability during different standing tasks. RMS of the angular velocities at the trunk and ankle levels increases as the task complexity increases. These results highlighted the relation of the level of PNP with postural strategies and instability.
de Bruin et al., 2012 [41]	29 DPN (with and without PNP) (61.9 ± 5)	1 accelerometer (DynaPort Mini-Mod, McRoberts BV) (ACC) Freq: not reported	Lower back	Walking at preferred velocity under two conditions. Single task: walking on the walkway; dual task: walking on the walkway with a counting task. The walkway contained a paved trajectory, cobble stones, and gravel rocks	Step time, step length, velocity, cadence	Significant differences between single versus dual task walking at baseline were identified for all gait parameters. Gait speed, step length, and cadence were significantly decreased under dual tasking, and step duration was significantly increased compared to normal walking. Gait speed, cadence, step duration, and step length under more challenging conditions can be reliably measured in adults with diabetes
Najafi et al., 2010 [43]	17 DPN (59.2 ± 8) 21 Healthy controls (24.4 ± 1)	2 Inertial sensors (ACC, GYR and MAG) (BalanSens™, Biosensics) Freq: not reported	1 Shank Lower back	Double leg stance for 30 s with open (EO) and closed eyes (EC) and feet together, with firm and foam surfaces.	COM sway area, hip and ankle motions	DPN individuals exhibit significantly greater COM sway than healthy subjects during both EO and EC conditions. Sway area was significantly higher than healthy subjects on average by 98%. No significant difference was observed for both ankle and hip sways during EO. At EC, both ankle and hip sways were significantly higher in DPN subjects. Results suggest that postural compensatory strategies during EO condition is significantly better in healthy subjects compared to DPN subjects. During EC condition, although postural control strategy was better in healthy subjects, the observed difference was not significant. It has been shown that PNP significantly affects postural compensantory strategies.

ACC: accelerometer; AP: anterior-posterior; CIDN: chronic inflammatory demyelinating polyneuropathy; CIPN: chemotherapy-induced peripheral neuropathy; COG: center of gravity; COM: center of mass; DFU: diabetic foot ulcer; DM: diabetes mellitus; DPN: diabetic peripheral neuropathy; **Freq**: sample frequency; GYR: gyroscope; MAG: magnetometer; ML: medio-lateral; NeP-DPN: neuropathic pain diabetic neuropathy; PNP-LL: peripheral neuropathy of the lower limbs; TUG: timed up-and-go test.

**Table 2:** Summary of the major characteristics of the PD reviews that met the inclusion criteria.

REFERENCE	REVIEW CHARACTERISTICS	NUMBER OF STUDIES INVESTIGATING PD	SAMPLE SIZE (H&Y Stage)	SENSORS (Number and Type)	EXTRACTED PARAMETERS
Morgan et al., 2020 [21]	Analysis of gait during home assessment	65 papers	Almost half of the studies used between 10 and 49 PD participants. 12 studies used fewer than 10 and 8 more than 100 participants.	45.5% of the studies used 1 sensor at the lower back; 2 studies used 3 sensors at lower back and feet; 1 paper used 1 sensor on the chest, 1 used 1 sensor on the wrist. 2 papers do not discribe the position	Features not specified.
Ghislieri et al., 2019 [27]	Analysis of standing balance	14 papers	From 10 to 58 PD patients (and one study with 104 patients)	The 93% of studies used 1 sensors on the lower back. 1 study used 3 sensors: 1 on the lower back and 2 on lower limbs	Jerk index, sway amplitude, range of acceleration signals, frequency dispersion and centroidal frequency.
Rovini et al., 2018 [22]	Analysis of gait during home assessment	30 papers	Ranging from 1 to 75 PD patients	6 papers (28.2%) used 1 sensor: 4 on the waist and 2 on the lower back. 10 (33.3%) papers used 2 sensors: 5 on the wrists, 1 on the feet, 3 on the ankles, one on ankle and dominant leg. 6 studies used 3 sensors on the waist and feet. 2 papers used 5 sensors (on wrists, ankles and trunk; on shanks, wrists and sternum). The last 3 papers used more than 6 sensors.	Average time and distance walked, cadence, gait speed, step length, swing time, double support time; stride time and stride time variability. Inter-trial variability, inter-subject variability; inter-task variability. Number of turns per hour, turn angle amplitude, turn duration, turn mean velocity, number of steps per turn, hourly frequency of turning, duration of each turn, number of steps per turn, peak and average rotational turning rate, jerk, variability of these measures throughout the day and week.
Merola et al., 2018 [52]	Analysis of gait and balance	6 papers	From 6 to 40 (and 2 studies with 190 and 139 PD patients)	Not reported	**Gait:** temporal (reaction time, gait cycle duration), spatial (step length, step height) and biomechanical (ankle torque, vertical landing force) variables, and gait strategies (i.e., number of steps, single versus multiple step response). **Balance and postural instability:** trajectory of the center of pressure (COP) and center of mass (COM) misplacement, trunk acceleration and postural sway
Vienne et al., 2017 [25]	General analysis of gait	16 papers	Not reported	11 studies (68.7%) described the assessment of PD with 1 sensor at the lower back. one paper used one sensor at one ankle, one at one shank and one at one foot. One paper used 2 sensors (upper and lower back), and one paper utilized 3 sensors at lower back and shanks	Features not specified.
Rovini et al., 2017 [53]	Analysis of wearable sensors on support of PD treatment and diagnosis	80 papers	From 5 to 47 (and 1 study of 75 PD patients)	Not reported	Statistical (e.g., mean, variance, skewness, kurtosis), frequency (e.g., energy, power spectral density, fundamental frequency), and spatiotemporal/kinematic (e.g., stride length, TUG time, stride velocity) features; step or stride segmentation.
Godinho et al., 2016 [16]	Mobile health technology characteristics	76 papers	Not reported	Not reported	ISway measures (jerk, RMS amplitude and mean velocity from the time-domain measures, and centroidal frequency); gait parameters with a high degree of accuracy; total number of walking bouts, the percent of time spent walking, the total number of steps, median walking bout duration, median number of steps, and median cadence per bout. Quality-related sensor derived measures included: frequency measures, regularity measures and the harmonic ratio.
Del Din et al., 2016 [23]	Analysis of gait during home assessment	19 papers	From 2 to 169 PD participants (and one study of 467 patients)	9 studies (47.3%) used 1 sensor on lower back; 3 used 2 sensors on thighs; 2 papers used 2 sensors on feet; 1 on both shanks and 1 used 1 sensor on the chest; the other papers used more than 4 sensors.	Number of walking bouts, walking duration, total number of steps, median number of steps per bout, bout duration, cadence, step and stride regularity, frequency domain measures (harmonic ratio, amplitude, slope and width of dominant frequency), step duration, step symmetry, acceleration range and dynamic stability
Oung et al., 2015 [50]	Assessment of motor disorders in PD	Not reported	Not reported	Not reported	Step frequency, stride length, entropy and arm swing
Hubble et al., 2015 [28]	Analysis of standing balance and walking stability	26 papers	From 5 to 67 PD patients	20 studies (76.9%) used 1 sensor on the lower back (sacrum/L3/L4/L5); 2 studies used 2 sensors on the shanks; 2 studies used 1 sensor on sternum/chest; 1 study utilized one sensor on the wrist; and another one on the lateral side of the pelvis.	Sway velocity (23% of studies), RMS accelerations (19% of studies) and jerk (19% of studies). Harmonic ratio (31% of studies) and stride time variability (27% of studies).
Steins et al., 2014 [51]	Assessment of functional activities with wearable devices	6 papers	Not reported	Not reported	Stride length, stride velocity, cadence, and turning velocity
Maetzler et al., 2013 [26]	Quantitative objective assessment of gait and balance	16 papers	Not reported	**Gait:** 4 papers used one sensor on the lower back (44.4%). 2 papers utilized 1 sensor on the shank and 2 papers 2 sensors on both feet. 1 paper used 1 sensor on the forearm and two studies used more than 5 sensors. **Balance:** 5 papers used 1 sensor on lower back (100%).	**Gait:** Phase coordination index of gait cycle; stride length; frequency-based measures of gait (harmonic ratio, amplitude, slope and width of dominant frequency); cadence, step time variability; peak trunk horizontal velocity, turning duration, turn-to-sit duration; time- and amplitude-based measures of sit-to-stand and stand-to-sit; peak trunk rotation velocity and rotation range of motion, turning velocity; Walk peak roll velocity, total turning duration, turn peak yaw and roll velocity. **Balance:** Velocity, jerk, acceleration, frequency-based measures; displacement, velocity; Peak trunk acceleration during anticipatory postural adjustments towards the stance leg; Hilbert-Huang transformation of postural parameters
Horak et al., 2013 [49]	Biomarkers of gait and balance	Not reported	Not reported	Not reported	**Gait:** Stride Time Variability, double support time, peak arm velocity, trunk rotation, gait velocity, cadence, stride length. Balance: Postural sway (area, velocity, frequency) and jerk.

## Abbreviations

ACC	Accelerometer
AP	Anterior-posterior
BBS	Berg Balance Scale
CFF	Cross-correlation function
CIDN	Chronic inflammatory demyelinating polyneuropathy
CIPN	Chemotherapy-induced peripheral neuropathy
COG	Center of gravity
COM	Center of mass
COP	Center of pressure
CV	Coefficient of variation
DFU	Diabetic foot ulcer
DM	Diabetes mellitus
DPN	Diabetic peripheral neuropathy (DPN)
EC	Closed eyes
EO	Open eyes
FAB	Fullerton Advance Balance test
Freq	Sample frequency
GYR	Gyroscop
HC	Healthy controls
iTUG	Instrumented, timed up-and-go test
MAG	Magnetometer
MeSH	Medical Subject Headings
ML	Medio-lateral
NeP-DPN	Neuropathic pain diabetic neuropathy
PD	Parkinson ’s Ddisease
PNP	Peripheral neuropathy
PNP-LL	Peripheral neuropathy of the lower limbs
PNP-PD	Peripheral neuropathy in Parkison disease
PNS	Peripheral nervous system
PRISMA	Preferred Reporting Items for Systematic Reviews and Meta-Analyses
RMS	Root mean square
SOT	Sensory organization test
TBS	Tinetti Balance scale
TUG	Timed up-and-go test

## Appendix A. Search Query

(1) Pubmed

- PN+wearable with MeSH

((Peripheral Nervous System Diseases[Mesh]) AND (wearable sensor*[Title/Abstract] OR wearable [Title/Abstract] OR mobile health technology[Title/Abstract] OR technology assessment[Title/Abstract] OR body-worn sensor*[Title/Abstract] OR portable device[Title/Abstract] OR inertial sensor[Title/Abstract] OR inertial measurement unit[Title/Abstract] OR acceleromet*[Title/Abstract] OR gyroscope[Title/Abstract] OR angular velocity[Title/Abstract] OR acceleration[Title/Abstract]) AND (mobility[Title/Abstract] OR gait[Title/Abstract] OR balance[Title/Abstract] OR postural balance[Title/Abstract] OR postural stability[Title/Abstract] OR postural strategies[Title/Abstract])).

- PD+wearable with MeSH terms

((Parkinson Disease[Mesh]) AND (wearable sensor*[Title/Abstract] OR wearable [Title/Abstract] OR mobile health technology[Title/Abstract] OR technology assessment[Title/Abstract] OR body-worn sensor*[Title/Abstract] OR inertial sensor[Title/Abstract] OR inertial measurement unit[Title/Abstract] OR acceleromet*[Title/Abstract] OR gyroscope[Title/Abstract]) AND (mobility[Title/Abstract] OR gait[Title/Abstract] OR balance[Title/Abstract] OR postural balance[Title/Abstract])).

(2) Scopus database

- PN+wearables

TITLE-ABS-KEY (“peripheral neuropath*”) OR TITLE-ABS-KEY (polineuropath*) OR TITLE-ABS-KEY (“small fiber neuropathy”) AND TITLE-ABS-KEY (“wearable sensor*”) OR TITLE-ABS-KEY (wearable*) OR TITLE-ABS-KEY (“mobile health technolog*”) OR TITLE-ABS-KEY (“technology assessment”) OR TITLE-ABS-KEY (“body-worn sensor*”) OR TITLE-ABS-KEY (“inertial sensor*”) OR TITLE-ABS-KEY (“inertial measurement unit*”) OR TITLE-ABS-KEY (accelerometer*) OR TITLE-ABS-KEY (gyroscope*) OR TITLE-ABS-KEY (angular velocity) OR TITLE-ABS-KEY (acceleration) AND TITLE-ABS-KEY (mobility) OR TITLE-ABS-KEY (gait) OR TITLE-ABS KEY (balance) OR TITLE-ABS-KEY (“postural balance”) OR TITLE-ABS-KEY (“postural stability”) OR TITLE-ABS-KEY (“postural strategies”).

- PD+wearables

TITLE-ABS-KEY (“parkinson’s disease”) OR TITLE-ABS-KEY (“parkinson disease”) OR TITLE-ABS-KEY (Parkinson*) AND TITLE-ABS-KEY (“wearable sensor*”) OR TITLE-ABS-KEY (wearable*) OR TITLE-ABS-KEY (“mobile health technolog*”) OR TITLE-ABS-KEY (“technology assessment”) OR TITLE-ABS-KEY (“body-worn sensor*”) OR TITLE-ABS-KEY (“inertial sensor*”) OR TITLE-ABS-KEY (“inertial measurement unit*”) OR TITLE-ABS-KEY (accelerometer*) OR TITLE-ABS-KEY (gyroscope*) AND TITLE-ABS-KEY (mobility) OR TITLE-ABS-KEY (gait) OR TITLE-ABS-KEY (balance) OR TITLE-ABS-KEY (“postural balance”).

(3) Web of Science database

- PN+wearables

TS = (“peripheral neuropath*” OR polineuropath* OR “small fiber neuropathy”) AND TS = ( “wearable sensor*” OR wearable* OR “mobile health technolog*” OR “technology assessment” OR “body-worn sensor*” OR “inertial sensor*” OR “inertial measurement unit*” OR accelerometer* OR gyroscope* OR “angular velocity” OR acceleration) AND TS = (mobility OR gait OR balance OR “postural balance” OR “postural stability” OR “postural strategies”).

- PD+wearables

TS = (“parkinson’s disease” OR “parkinson disease” OR Parkinson*) AND TS = (“wearable sensor*” OR wearable* OR “mobile health technolog*” OR “technology assessment” OR “body-worn sensor*” OR “inertial sensor*” OR “inertial measurement unit*” OR accelerometer* OR gyroscope*) AND TS = (mobility OR gait OR balance OR “postural balance”).
